# Hybrid aerodynamic and structural optimization of super-tall buildings under wind loads for sustainable and cost-efficient design

**DOI:** 10.1038/s41598-026-45932-0

**Published:** 2026-03-30

**Authors:** Aiman H. H. Al-Masoodi, Nasir Shafiq, Abtisam Hasan Hamood Al-Masoodi

**Affiliations:** 1 Department of Civil and Environmental Engineering, Universiti Teknologi PETRONAS (UTP), Perak, Malaysia; 2 Department of Physics, Faculty of Applied Science, Hajjah University, Hajjah, Yemen

**Keywords:** Computational methods, Genetic algorithms, Structural optimization, Super-tall buildings, Wind-resistant design, Sustainability, Energy science and technology, Engineering

## Abstract

Recently, there has been advancement in tall building design to achieve lighter structures, which led to an increase in the challenges that are related to wind resistance and cost efficiency. This study is to provide multi-optimization gathering between the aerodynamic optimization with a radial basis function (RBF)-based design and structure optimization by utilizing a genetic algorithms (GAs) developed by an enhanced penalty function. Subsequently, the optimized wind loads are employed to optimize the structure weight while ensuring resilience against wind-induced loads. The findings indicate significant enhancements, featuring a 28.2% drop in maximum top displacement accompanied by just a 0.76% reduction in total gross floor area for the optimal corner configuration (chamfered). This approach enhanced the wind load due to optimal corner configurations for structural optimization of the building, leading to redistribution of lateral structural sections of the building along the height to minimize the weight with compliance of required performance building criteria such as displacement, drift, and acceleration constraints simultaneously. The output of the optimization achieved 28.8% less concrete, leading to a reduction of 4630 tons of embodied CO_2_ emissions. Overall, the proposed hybrid framework effectively enhances building performance, sustainability, and economic viability in the design of super-tall buildings.

## Introduction

In the current century, there is a trend to construct super-tall buildings due to a shortage of land, which makes it more expensive, and to be an attractive landmark for the city around the world. Therefore, this desire creates many challenges, especially wind effects, complicated engineering design, sustainability considerations, and construction intricacies^1^. This trend is fueled by escalating urban populations, limited urban space, and the rising value of urban land, which has increased the economic appeal of high-rise structures. Consequently, there is a growing imperative to maximize land use efficiency in densely populated areas, where suitable construction sites are both scarce and expensive. Addressing these issues necessitates a focus on wind mitigation strategies and structural optimization techniques^2–5^, which are crucial for minimizing construction costs and ensuring the viability of such ambitious architectural projects.

Nowadays, integrating computational fluid dynamics (CFD) with parametric modeling, optimization algorithms, and finite element analysis has become an efficient solution for addressing aerodynamic challenges in tall building design^6,7^. Thus, designers are able to explore and evaluate the building’s shapes more accurately to determine the optimum aerodynamic design of the building. Corner modification, such as chamfering, rounding, and recessing, is one of the important techniques that have been used to optimize the aerodynamic effect for super-tall buildings, which has a small impact on the building configuration^8–10^. Based on tunnel test experiments done by Kwok^11^, by utilizing aeroelastic and high-frequency force balance, it was reported that the corners with chamfered configuration and horizontal slots have a significant influence on building response due to both along-wind and across-wind forces. In the previous study, it was found, by utilizing CFD analysis, that rounded corners are highly effective in mitigating the wind effect^12^. Based on the previous investigation, it is proved that chamfered and rounded corners are more effective to mitigate the wind effect, which changes the layout configuration slightly as investigated by subsequent experimental studies^13–16^. Also, the recessed corners have been investigated and found that it is most effective modification for aerodynamic performance and financial cost^8,17^. Furthermore, one of the research has been studied through wind tunnel tests, which reported that recessed corners are able to reduce the aerodynamic performance^18^. While previously, the study depended mainly on experimental approaches^11,19^, recent research relies on CFD to evaluate and optimize aerodynamic performance^7,20^.

Artificial intelligence (AI) has improved human lives and solved real-world problems alone and in combination^21,22^. Genetic algorithm (GA), developed by John Holland in 1975^23^, is widely used in engineering. GAs have optimized reinforced concrete flat slab buildings^24^, tunnel profiles in different ground conditions^25^, steel beam cross-sections^26^, and arch bridges^27^. GAs for tall building optimization have received little research. These efforts aim to optimize to provide sustainable designs. It was found that there is a reduction in structure weight due to corner modification, leading to a reduced wind response^2^. Also, another study reported that structural optimization by utilizing GAs can reduce the structural element and various concrete compressive strengths to minimize cost and CO_2_ emissions, resulting in sustainable designs^28^.

Finite element analysis (FEA) is a numerical method used to tackle complex engineering problems, and it originated from early efforts to simplify complicated systems^29^. Currently, FEA is one of the vital tools that has been used by the engineers and researchers to simulate the real project for enhancing the optimization for material cost efficiency and structural stability, as well as enhancing the performance design and safety for the project^30^. Furthermore, as computational power keeps improving, FEA is evolving too, providing better accuracy and efficiency in tackling modern engineering challenges. Today, it is one of the most important tools for engineers and scientists, allowing for advanced simulations and optimizations in various fields^29^. Furthermore, when the GA is integrated with FEA for analyzing the elements in the structure, it enhances the accuracy and structural element optimization in huge projects such as super-tall buildings^31,32^. GAs are capable of providing constraints for design criteria to improve the FEA analysis to make sure the optimum solution is safe and performs well. The integration between GAs and FEA is very important to provide the best, sustainable, and cost-effective design^31^.

Engineers need to be very careful with lateral drift and top acceleration when designing tall buildings that can withstand wind. This is to make sure they are stable and comfortable. It’s hard to find a balance between structure serviceability, cost-effectiveness, and safety^1,2,4,33^. For safety, tall buildings must be able to withstand wind loads without losing their structural integrity. Serviceability design ensures that the structure maintains its functionality and structural integrity under wind loading by controlling deformations, accelerations, and vibrations within acceptable limits. Occupant comfort is one important aspect of serviceability, but it also includes preventing structural and non-structural damage. To be cost-effective, solutions must be able to be made without putting safety or serviceability at risk. To meet all of their needs, tall buildings need new designs, new materials, and a lot of research.

In addition to aerodynamic and structural optimization of tall buildings, numerous studies focus on optimal design to determine control strategies that reduce dynamic response. Additionally, some devices, such as tuned mass dampers and viscous dampers, are used for multi-optimization and have proven effectiveness in minimizing wind and seismic vibrations^34–38^. The literature reports that the installation of tuned mass dampers in offshore structures and tall buildings subjected to seismic effects has significantly reduced structural vibrations, thereby enabling optimal geometry and structural member sizes^34–36,39^. Chan et al. presented a review of the installation of multiple tuned mass dampers to reduce vibration in high-rise buildings subjected to wind or seismic loading^35^. Whittle et al. discussed placement techniques for five viscous dampers to control seismic loading effects on a tall building^39^; similar findings are reported in other studies^36–38^. However, the present study differs in that it focuses on the coupled aerodynamic–structural optimization of super-tall buildings without introducing supplemental control devices, aiming to achieve inherent wind-resilient and cost-efficient structural weight.

In this paper, a novel integration of a multi-objective computational approach for aerodynamic and structural optimization is proposed, which has not been addressed together in the previous study. Aerodynamic optimization is done by the verified CFD system, which integrates with the parametric model and FEA to evaluate aerodynamic performance. Furthermore, in structural optimization, GAs is developed to optimize the lateral resistance system of the super-tall building in compliance with static and dynamic performance criteria simultaneously. Minimum material consumption and wind load optimization result in a cost-effective, environmentally friendly design that enhances structural sustainability and stability.

## Enhancing research reliability and applicability

To strengthen this study, a validated CFD model is used, which is applied to existing buildings for evaluating the building performance at real-world conditions, which represent the complexities and unique characteristics that engineers face on actual projects.

### CFD verification with empirical results of the CAARC building

**Fig. 1:**
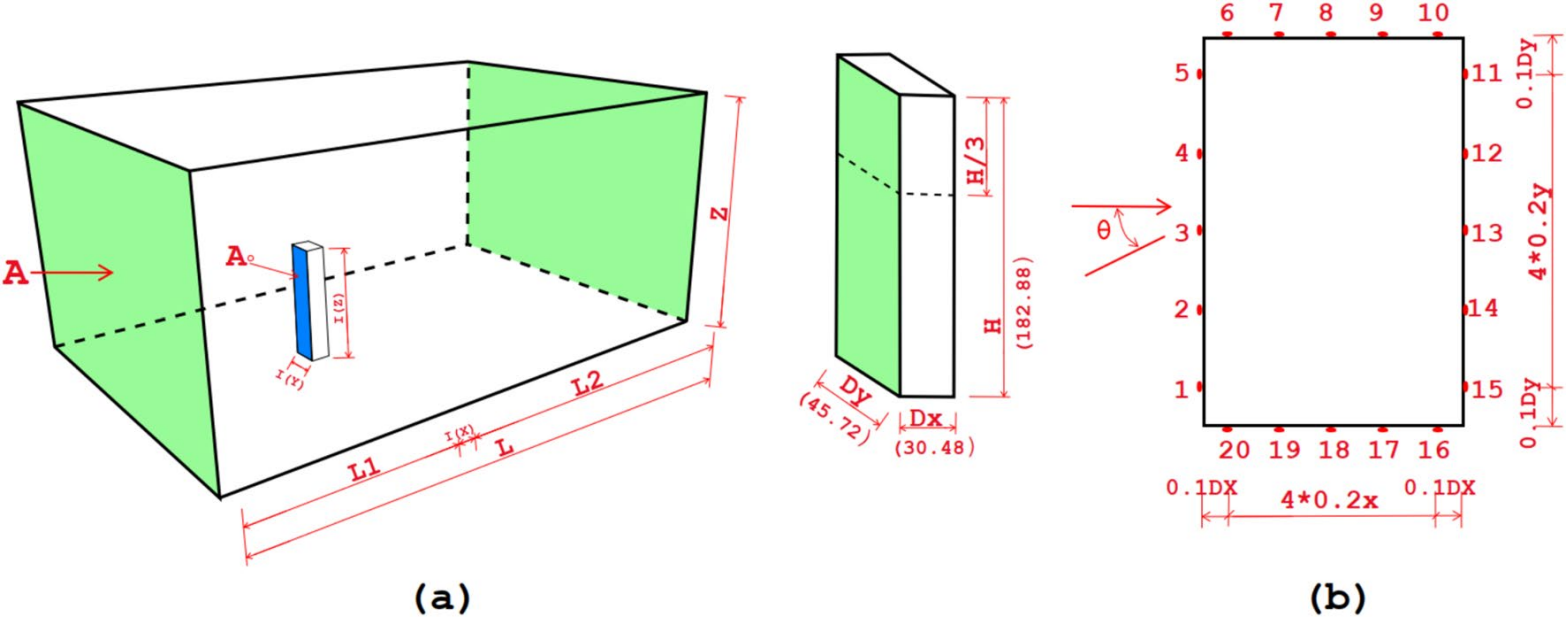
(**a**) Settings of the computational domain dimensions of the tunnel model, (**b**) Measuring points of tap pressure at 2/3 of the height^6^.

The validation of the numerical CFD model employed in this research was corroborated against the benchmark established by the Commonwealth Advisory Aeronautical Research Council (CAARC) for tall building models. The verification method was derived from the authors’ prior research on the aerodynamic performance of tall buildings using a computational methodology as shown in Fig. 1^6^. The computational results in that study were validated by empirical wind tunnel data from Tongji University (TJ) at a 0° wind direction, demonstrating significant concordance in the mean wind pressure coefficient (Cp), with variances restricted to ± 15%, as indicated by^40^.

**Fig. 2:**
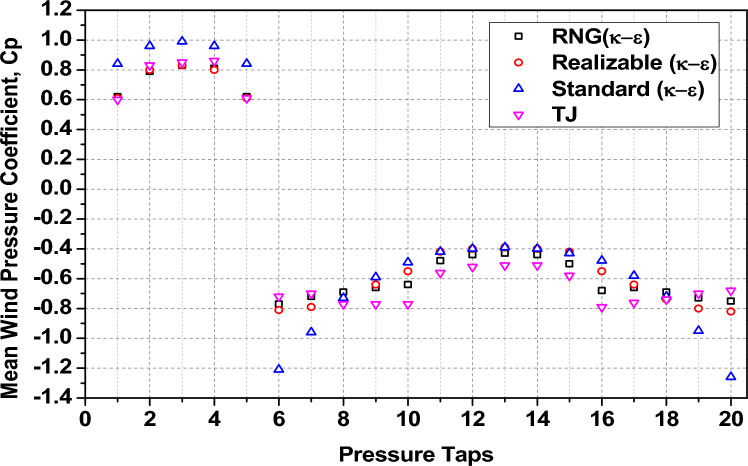
Cp readings for varying turbulence models with TJ experimental results^6^.

The numerical domain, blockage ratio (less than 5%), and boundary conditions were acknowledged in accordance with CAARC recommendations^6,40^, ensuring alignment between the CFD model and the empirical configuration. We used the RNG (k–ε) turbulence model, the SIMPLE pressure–velocity integration method, and the second-order upwind discretization. We also changed the mesh grid to make sure that the near-wall resolution was correct. The verification showed that the average difference from the TJ empirical findings was 9.3%, as shown in Fig. 2. This shows that the turbulence model and solver configuration used were appropriate.

Therefore, the current CFD configuration was recognized based on this validated model to ensure the dependability and uniformity of the aerodynamic performance findings presented in this work. The approach and parameters match with those employed and checked in previous published studies, which support this study expansion.

### Application to an existing building case study

**Fig. 3:**
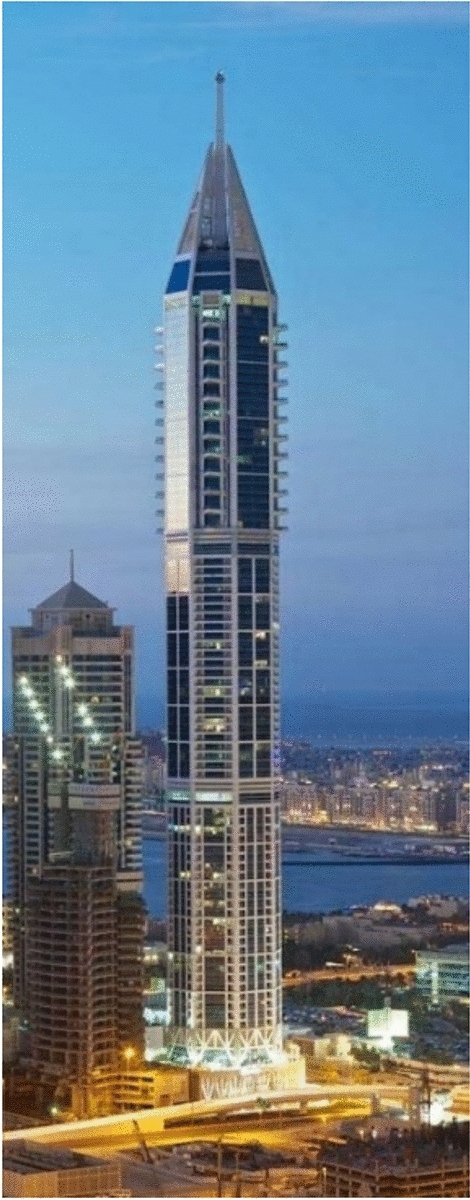
Marina Tower, Dubai.

An existing project was chosen to implement the proposed optimization methods and prove their liability and accuracy, which presented 23 Marina, an 88-story residential supertall building in Dubai, United Arab Emirates, with a height of 392.8 m (1289 ft), the 6th tallest residential building in the world, as shown in Fig. 3. It was carefully chosen because of its octagonal geometry, which can be effective for corner modification investigation in this study by using a constant typical floor with a height of 360 m. The loads that were assumed for this case study are the gravity forces such as self-weight, superimposed dead loads (4 kPa, including brick walls and M&E services), and a live load of 1.5 kPa for residential occupancy as stipulated in relevant design standards. In addition, the wind loads are generated from CFD analysis and applied to the structural model. Furthermore, it presents a challenging exercise to incorporate minor aerodynamic modifications that maintain the intended purpose of the architectural form and structural design to further mitigate wind effects with a less expensive and practical computational method to evaluate tall buildings’ responses to wind-induced motions at the preliminary design stage. Therefore, with its octagonal shape and vertical and slender height, this project presents an appropriate case study that falls within the scope defined by this research. For the sake of this research, CFD optimization is firstly implemented to find the optimal corner shape with respect to wind-induced load and overall structural response. Then GA optimization is implemented to minimize the structural weight of the building while maintaining its static and dynamic responses within allowable limits. For clear understanding, Table 1 specifies the design variables, objective functions, and constraints adopted in the optimization framework for the case study. This case study offers a realistic and practical basis for evaluating aerodynamic corner modifications for super-tall octagonal buildings using a computational approach.

**Table 1:** Summary of optimization framework for the case study.

Category	Description
Design variables	Aerodynamic corner modification parameters; structural member sizing variables
Objective functions	Minimize top displacement; minimize total structural weight
Constraints	Maximum top deflection; inter-story drift limits; Top acceleration

## Wind-performance optimization procedure

### Wind load simulation

For this research, the super-tall building was modeled as a separate structure within the CFD computational tunnel, without the effect of the surrounding buildings. It was conducted for the wind load with a speed of 30 m/s, which represents the typical wind conditions surrounding the super-tall building. It was assumed in this simulation that there was steady and incompressible flow, with using RNG k-ε turbulence model used to reflect the turbulence impact around the building. It was employed with a fine mesh to ensure better resolution and capture of flow characteristics. The wind load was applied at a 0° direction to represent the wind effect for all the directions around the building due to its octagonal shape. This setup is to show a realistic behavior of the wind application, which is important for performing evaluation for the optimization process addressing key parameters such as drag, lift, deflections, and torsional moments to enhance the aerodynamics based on the structural performance of the building.

### Parametric computational process for aerodynamic performance

**Fig. 4:**
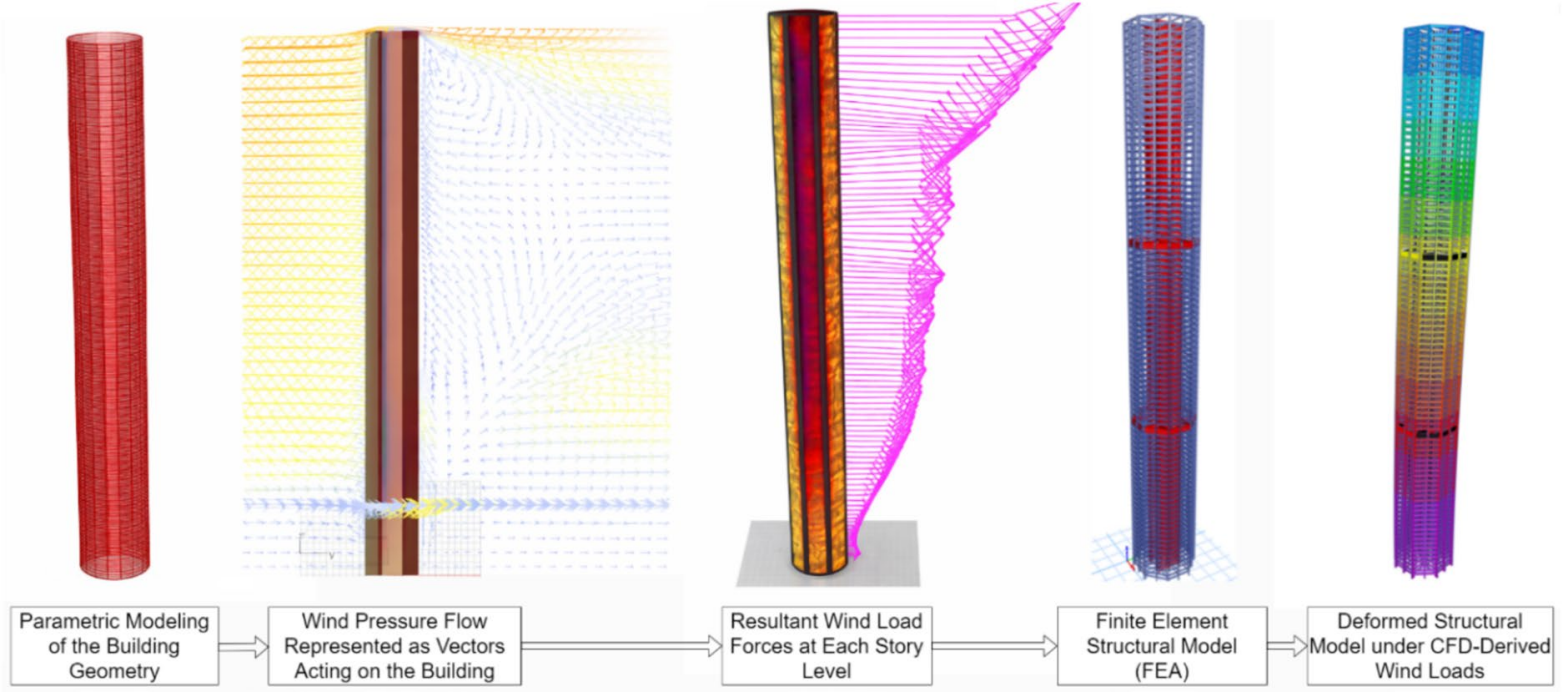
CFD computational model for wind simulation and structural response.

This study adopted a computational approach that gathered a parametric model that was linked to the CFD solver, which interfaces with OpenFOAM. Subsequently, a structural model was constructed, integrating fluid–structure interaction (FSI) to simulate wind pressures derived from the parametric model as dynamic forces during the optimization phase. The determination of the tunnel domain dimensions was based on the building’s height (H), resulting in dimensions of approximately 2.3 m for the windward, sides, and top, and 10 times the height (10H) for the leeward side^41^. The blockage ratio was computed at 3.54%, well below the 5% threshold^6,40,42^. It was determined to have aerodynamic optimization with acceptable corner modifications, so it was developed by using Python between the computational methods to transfer the output stress of the wind to vectors of force with moment for each floor of this super-tall building. The FSI approach adopted in this study is a one-way coupling procedure, where aerodynamic loads obtained from CFD simulations are transferred to the structural model without feedback to the flow field. Then, an FEA 3-D model using ETABS was modeled, consisting of high-strength concrete columns (Grade G60) and Grade G50 concrete for other structural members. The analysis has been performed in each round of this optimization cycle to assist the structures for wind response as illustrated in Fig. 4.

### The surrogate RBF optimization technique

The surrogate RBF optimization technique is used to create the aerodynamic optimization process. This technique uses a radial basis function and combines all prior solvers and coupling into an optimization algorithm. It is cost-effective approximations instead of expensive structural assessments that are easy to evaluate. These models depend on a small number of accurate response evaluations to create an estimated surface that matches the genuine function response, which is the objective function in a certain design space^43^. This study selected the Multiquadric Radial Basis Function (RBF) as the most appropriate surrogate model, based on several benchmark studies concentrated on optimization in architectural contexts^44^.

#### Design of experiment (DOE)

**Fig. 5:**
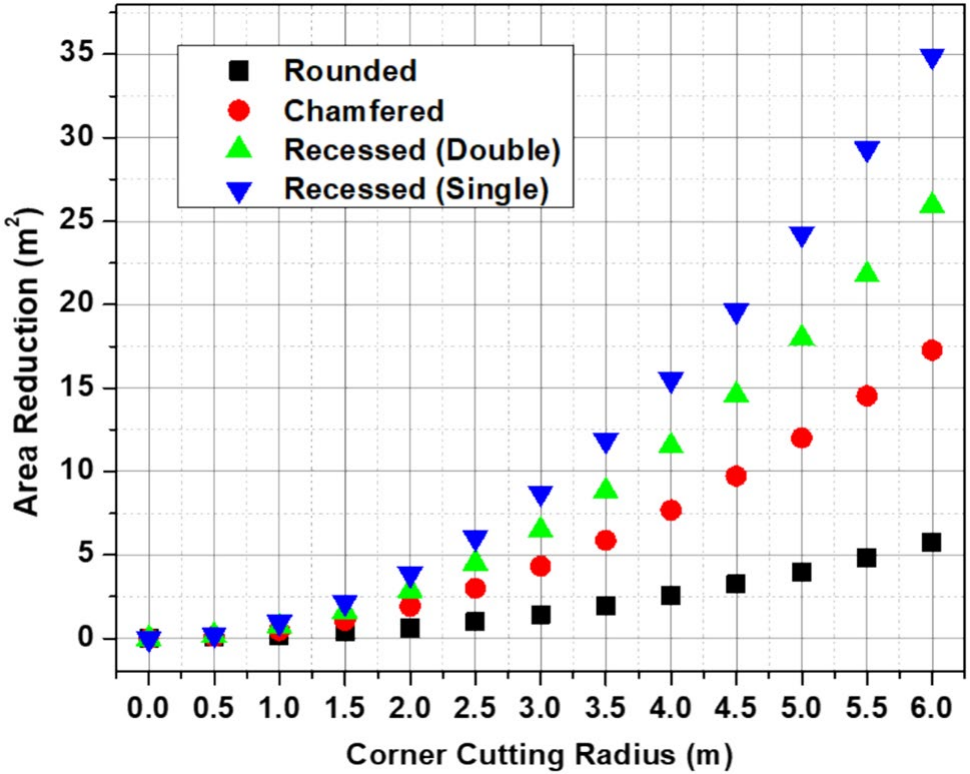
Sampling points in different corner modification types.

It is essential to understand that the amount and quality of the sample used to construct the surrogate model affect how well it works and how accurate it is. The surrogate should be made with as much information as feasible to make accurate forecasts. The sampling points used to fit the surrogate can show this information. But it’s important to find a balance when it comes to the number of sampling points. More points make the model more accurate, but they also make it more expensive to build and test the model. Two factors are usually used to make the sample points: corner cutting radius and area reduction. The corner cutting radius variable was made in a way that all the points had the same size. The area reduction is based on the corner cutting radius. As shown in Fig. 5, there are 13 sample points in the design space for the four types of corner modifications.

#### Objective function

The goal of the objective function is to make the structure work better when it is loaded by wind. So, in this study, the goal is to come up with an objective function that would reduce the total top displacement (mm) produced by wind forces for different types of corner modifications. On the other hand, the objective function is likewise set up to lower the maximum resulting displacement at the top of the structure. This study establishes a two-dimensional design space whereby each design point $i=1, 2, 3,\dots,N$ signifies a distinct combination of the two design variables, $a$ and $b$, which pertain to the corner cutting radius and the area reduction associated with the corner modification. For a building with $j=1, 2, 3,\dots,M$ stories, the objective function can be mathematically expressed for minimizing the total top displacement as:

$$f\left( {a_{i} , b_{i} } \right) = \delta_{Top}$$

Subject to.

$$\Delta_{j} \le \Delta_{limit}$$, $$a_{i}^{L} \le a_{i} \le a_{i}^{U}$$, and $$b_{i}^{L} \le b_{i} \le b_{i}^{U} ,$$

where $f\left({a}_{i}, {b}_{i}\right)$ is the predicted RBF value for the total top displacement ${\delta }_{Top}$, ${\Delta }_{j}$ is the maximum story drift value and ${\Delta }_{limit}$ limit is the permitted limit according to the wind-drift standard. This study uses the Eurocode requirement of H/500 as the limit for drift. The parameters L and U show the lowest and highest values for the design variables, respectively.

## Structural optimization using GAs

This part is to explain the GAs approaches to optimize the super-tall building under wind-induced loads, which aim to minimize the structural weight with respect to the safety requirements and serviceability constraints. For this procedure, consider the main constraints, which are top displacement, inter-story drift, and top acceleration, which are employed by GAs to opt for the structural weight based on these limitations.

### Structural optimization problem definition

Few studies have documented efforts to improve the structural performance of tall buildings in the face of wind excitations, which could lead to a decrease in the amount of materials used in construction while simultaneously increasing their serviceability and safety. Most of these studies have focused on seismic load effects.

#### Formulating objective functions by the GA

In structural design optimization, the weight of the structure is typically regarded as the goal function. In the context of a concrete tall building structure, the objective function can be articulated as the minimization of the weight of the structural lateral system concrete. Consequently, for a three-dimensional structural frame comprising I = 1, 2, 3, …, N types of core walls, columns, and beams, and j = 1, 2, 3, …, M stories, the goal function for wind resistance is articulated as:

To mitigate:

$$f\left( x \right) = \mathop \sum \limits_{i = 1}^{N} \rho A_{i} l_{i}$$

Limited to

$$b_{i}^{L} \le b_{i} \le b_{i}^{U} ;h_{i}^{L} \le h_{i} \le h_{i}^{U} ; t_{i}^{L} \le t_{i} \le t_{i}^{U}$$

$$b_{i} , h_{i} , t_{i} = S_{i} \in \left\{ S \right\},$$

where ρ denotes the density of concrete, ${A}_{i}$ represents the cross-sectional area of the i_th_ category of element section, and ${l}_{i}$ indicates the length of the ${i}_{th}$ type of element section. ${b}_{i}$ and ${h}_{i}$ denote the width and depth of the column/beam section in the ith kind of section, respectively, while ${t}_{i}$ represents the thickness of the core wall in the ${i}_{th}$ type of section. ${S}_{i}$ denotes the indices for the section sizes domain defined within $\{S\}$.

Eliminating vectors that violate constraints frees constrained optimization problems^30,45^. Objective functions that violate the constraint condition will be penalized by increasing their values above the target, reducing their fitness in the GA, and being eliminated by the genetic operators. Concurrent FEA and Python simulations address the displacement constraint in this experiment. Python starts wind-induced displacement FEA. Python’s constraint violation penalty function lowers objective function fitness. Minimizing objective function F(x) inequality constraint:

$$f\left( x \right)_{penalized} = f\left( x \right) + K\mathop \sum \limits_{k = 1}^{V} c_{k}$$

The penal term for constraint violations is ${c}_{k}$, the penalty coefficient is *K*, and the original goal function is $f\left(x\right)$ (minimizing structural weight). In constrained optimization problems, the easiest way to tackle optimization problems is with penalty functions, that penalize vectors which violate constraints^32^. Wind-resistant buildings have an advantage by reducing core walls, columns, and beams. Penalties for objective functions that violate constraint criteria increase their values beyond the goal, decreasing GA’s fitness. Due to fitness decline, genetic operators are eliminated.

#### Constraints in tall building design for wind resistance

To reduce building weight while meeting international optimization constraints like generated lateral inter-story drifts, top displacement, and acceleration^46,47^. Equations (1) and (2) establish the lateral displacement (${u}_{H}$ and ${v}_{H}$) and inter-story drifts (${u}_{j}$ and ${v}_{j}$) for both along-wind and across-wind, respectively, as primary design constraints, constraining their values within specified limits. Here, the diagonal vector of lateral displacement denotes the displacement limitation (δ_limit_), such as H/500 according to the EN 1991-1-4^47^. Additionally, the inter-story drift ratio for the ${j}_{th}$ floor regarding floor height ${z}_{j}$, and the relevant design code specifies a limited value for allowable inter-story drift (${\Delta }_{limit})$, which is about 0.005 times the story height (H)^48^. In Eq. (3), ${\ddot{x}}_{H}$, ${\ddot{y}}_{H}$ represent the top-story peak accelerations in the x and y directions as well as their limit in a practiced standard, and $x{\ddot{y}}_{H}$ represents the combined top-story peak acceleration in x–y directions, denoted by ${a}_{limit}$. Therefore, the constrain function in general formulation is stated by Eq. (4)

1 $$\frac{{u_{H} }}{H} \le \delta_{limit} ;\;\; { }\frac{{v_{H} }}{H} \le \delta_{limit} ;\;\; \frac{{\sqrt {u_{H}^{2} + v_{H}^{2} } }}{H} \le \delta_{limit}$$

2 $$\frac{{u_{j} - u_{j - 1} }}{{z_{j} - z_{j - 1} }} \le \Delta_{limit} ;\;\; \frac{{v_{j} - v_{j - 1} }}{{z_{j} - z_{j - 1} }} \le \Delta_{limit}; \;\; \frac{{\sqrt {\left( {u_{j} - u_{j - 1} } \right)^{2} + \left( {v_{j} - v_{j - 1} } \right)^{2} } }}{{z_{j} - z_{j - 1} }} \le \Delta_{limit}$$

3 $$\ddot{x}_{H} \le a_{limit } ;\;\; \ddot{y}_{H} \le a_{limit } ; \ddot{x}y_{H} \le a_{limit }$$

4 $$f_{k} \left( x \right) \le 0$$

As indicated by Eqs. (5), (6), and (7), addressing constraints for k = 1, 2, 3, … V constraints is crucial in optimization processes. However, GAs are typically designed to handle unconstrained optimization problems. To effectively manage constraints within this framework, it is necessary to adopt a straightforward method. It is common practice to transform a constrained optimization problem into an unconstrained one by applying penalizing vectors to the violated constraints^32^. As a result, the constraints were normalized.

5 $$\frac{{\frac{{u_{H} }}{H}}}{{\delta_{limit} }} - 1 \le 0;\;\; \frac{{\frac{{v_{H} }}{H}}}{{\delta_{limit} }} - 1 \le 0;\;\; \frac{{\frac{{\sqrt {u_{H}^{2} + v_{H}^{2} } }}{H}}}{{\delta_{limit} }} - 1 \le 0$$

6 $$\frac{{\frac{{u_{j} - u_{j - 1} }}{{z_{j} - z_{j - 1} }}}}{{\Delta_{limit} }} - 1 \le 0;\;\; \frac{{\frac{{v_{j} - v_{j - 1} }}{{z_{j} - z_{j - 1} }}}}{{\Delta_{limit} }} - 1 \le 0;\;\; \frac{{\frac{{\sqrt {\left( {u_{j} - u_{j - 1} } \right)^{2} + \left( {v_{j} - v_{j - 1} } \right)^{2} } }}{{z_{j} - z_{j - 1} }} }}{{\Delta_{limit} }} - 1 \le 0$$

7 $$\frac{{\ddot{x}_{H} }}{{a_{limit } }} - 1 \le 0;\,\, \frac{{\ddot{y}_{H} }}{{a_{limit } }} - 1 \le 0;\,\, \frac{{\ddot{x}y_{H} }}{{a_{limit } }} - 1 \le 0$$

All of the lateral frame sections can be identified by performing eigenvalue simulation according to the standards of ISO 10137:2007^49^ and ISO 6898^50^, as stated in formula Eq. (8). Additionally, to meet the safety and performance standards outlined in the international codes, it is necessary to verify that the acceleration is within the limits^31^.

8 $$\ddot{x}_{H} = \sqrt {2 {\mathrm{ln}} f_{1} T} + \left( {0.68 + \frac{\ln R}{5}} \right)\exp \left( { - 0.35 - 0.41\ln f_{1} } \right)$$

Within a return year period, ${\ddot{x}}_{H}$ is the maximum acceleration for T minutes. The observing time is usually set at 10 min, and R, which represents a 10-year return period^31^, f is the natural frequency of the first mode. $\sqrt{2 \mathrm{ln}{ f}_{1}T}$ is peak factor, ($0.68+\frac{\mathrm{ln}R}{5}$) is the adjustment factor, and the root-mean-square value of the natural frequency acceleration function is $\mathrm{exp}(-0.35-0.41\mathrm{ln}{f}_{1})$.

#### Fitness evaluation in GAs

To calculate the fitness value for each individual, the objective function must be modified by a penalty coefficient, C, due to the normalization of constraints (Eq. 9). This method guarantees that the optimization process complies with essential restrictions while efficiently minimizing structural components.

9 $$c_{k} = \left\{ {\begin{array}{*{20}l} {f_{k} \left( x \right)} \hfill & {{\mathrm{if}}\;\;f_{k} \left( x \right) > 0} \hfill \\ 0 \hfill & {{\mathrm{if}}\;\;f_{k} \left( x \right) \le 0} \hfill \\ \end{array} } \right.$$

where C represents ${\sum }_{k=1}^{V}{c}_{k}$. The modified objective function can then be expressed according to Eq. (10).

10 $$\emptyset \left( x \right) = g\left( x \right)\left( {1 + KC} \right)$$

, where $\varnothing \left(x\right)$ is the modified objective function, $f\left(x\right)$ indicates the true objective function, $C$ represents the violation coefficient, and $K$ is a penalty factor. Deflection restrictions are K = 10, and drift constraints are 1000 in this example study. Fitness must be calculated from objective functions. To calculate fitness in a minimization optimization problem, subtract $\varnothing \left(x\right)$ from a specified value. All fitness values should be positive, with those meeting the minimum having better fitness^51,52^. In order to calculate fitness, subtract the unconstrained objective function value ${\varnothing }_{i}\left(x\right)$ from the sum of the highest and minimum values in the population of individuals in the ${i}_{th}$ generation.

The fitness factor is calculated using Eq. (12). This approach efficiently translates goal function into a fitness metric, enhancing optimization. A population has n people. Figure 1 illustrates GA’s structural optimization.

11 $$F_{i} = [\emptyset_{i} (x)_{max} + \emptyset_{i} \left( {x)_{min} } \right] - \emptyset_{i} \left( x \right)$$

12 $$Fc_{i} = \frac{{F_{i} }}{{F_{avg.} }}\;\;{\mathrm{and}}\;\;F_{avg.} = \sum \frac{{F_{i} }}{n}$$

### Structural optimization approach

Several settings and parameters need to be set up in order to apply the created GA method to the examined case. The optimization algorithm, coding/decoding approach, and regulating parameters must be considered while formulating the examined problem.

#### Problem formulation

**Fig. 6:**
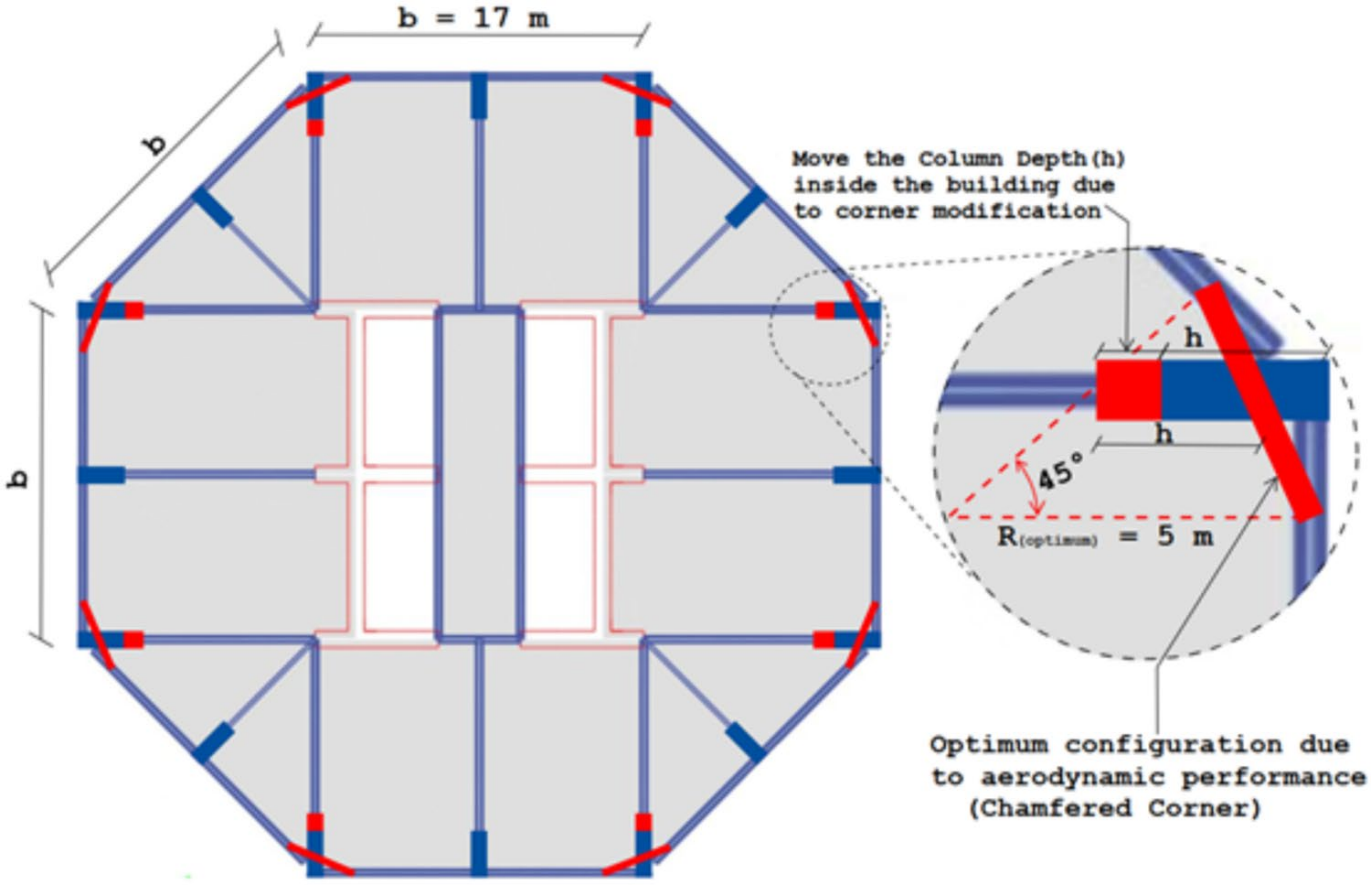
Structural plan of the case study illustrating the original layout and optimized chamfered-corner configuration.

Use 3D components to build core walls, beams, and columns. The lateral structural system is optimized, such as core walls, beams, and columns, so floors and internal beams are handled as constants for computation as mentioned in Fig. 6. This figure illustrates the structural plan of this case study, including the configuration of the original model and the optimum corner modification that was adopted to enhance the aerodynamic performance.

The building appears to be foundation-fixed. Every floor is 150 mm thick. Core walls, columns, and beams are separated into five equal portions depending on super-tall building height for realistic construction. Each level has the same-sized columns and beams. Table 2 shows FE model structural component dimensions. W1, W2, W3, W4, and W5 are the core walls of the 73rd-90th, 55th-72ed, 37th-54th, 19th-36th, and 1st-18th floors. In Table 2, C1, C2, C3, C4, and C5 are frame columns, and B1, B2, B3, B4, and B5 are floor frame beams. The original structural components were established based on engineering expertise and judgement in accordance with the Eurocode design guidelines and standards^53^.

**Table 2:** Structural section grouping.

Structural element	Category	Plain size	Minimum size	Maximum size
Core wall thickness (mm)	W1	850	425	1000
	W2	800	375	900
	W3	750	325	850
	W4	700	275	800
	W5	650	225	750
Column depth (mm) × Width (mm)	C1	2325 × 775	1900 × 635	2475 × 825
	C2	2275 × 760	1850 × 620	2425 × 810
	C3	2225 × 745	1800 × 600	2375 × 795
	C4	2175 × 725	1750 × 585	2325 × 775
	C5	2125 × 710	1700 × 570	2275 × 760
Beam depth (mm) × Width (mm)	B1	1225 × 410	800 × 270	1375 × 460
	B2	1175 × 395	750 × 250	1325 × 445
	B3	1125 × 375	700 × 235	1275 × 425
	B4	1075 × 360	650 × 220	1225 × 410
	B5	1025 × 345	600 × 200	1175 × 395

In 25 mm increments, lift core thickness, column, and beam depths were changed for construction efficiency. Table 2 reveals that columns and beams in the supplied groups were one-third the height to reduce design variables and computation time. Top displacement and inter-story drift design limitations were analyzed using Eurocode-2^54^. The highest deflection limit is 720mm, and the inter-story drift limit is 0.005 of the floor-to-floor distance for a 360 m super-tall building. ISO 10137:2007 specifies a top acceleration threshold of 0.21 m^2^/s and a one-year return time. These limits ensure equipment structural safety and comfort under dynamic wind and static loads throughout the building’s lifespan.

#### Algorithm implementation and data interpretation

Design variables in GAs can be represented using real and binary numbers. This research, however, employed a binary technique for its extremely efficient uniform crossover and enhanced computing efficiency by utilizing binary digits (0 and 1), as it pertains to the machine’s native language of 0 and 1. For the 15 design variables categorized by the designated groups, modifications to the sectional area of the design variables were scheduled within a range of 625 mm, allowing for a reduction or augmentation of 150 mm in section size, with adjustments made in 25 mm intervals. This will generate a set {S} of 32 sections of variance:

$$\{ {\mathrm{S}}\} = \left\{ {{65}0,\;{625},\;{6}00,...,{-}{75},\;{-}{1}00,\;{-}{125}} \right\}.$$

To generate a binary code for {S}, 5 bits are required for 32 binary representations, with (00000) denoting the first member in the set {S} and (11111) denoting the last element in the set {S}. Each of the 15 design variables (groups) is represented by a gene (substring) corresponding to a value from the {S} set and its binary encoding. All multi-design variable genes are subsequently interconnected to constitute a chromosome (string). Consequently, with 15 design variables, each substring comprising 5 bits, each individual solution is represented by a chromosome (string) with a length of 75 bits consisting of 0s and 1s.

#### GA control parameters for the structural optimization

Based on early tests showing convergence and time efficiency, therefore this GA had a fixed population and generation of 50. Elitism, crossover, and mutation will create generation 2. Mutations are 0.0017, and recombination crossovers are 0.7 per single-point crossing. Maximum displacement limitation penalty K1 is 10, and inter-storey drift ratio penalty K2 is 1000. Because the first trials took a long time and approached convergence, this GA set the population and generation to 50. Elitism, crossover, and mutation will be used to generate the next generation after the first is evaluated.

**Fig. 7:**
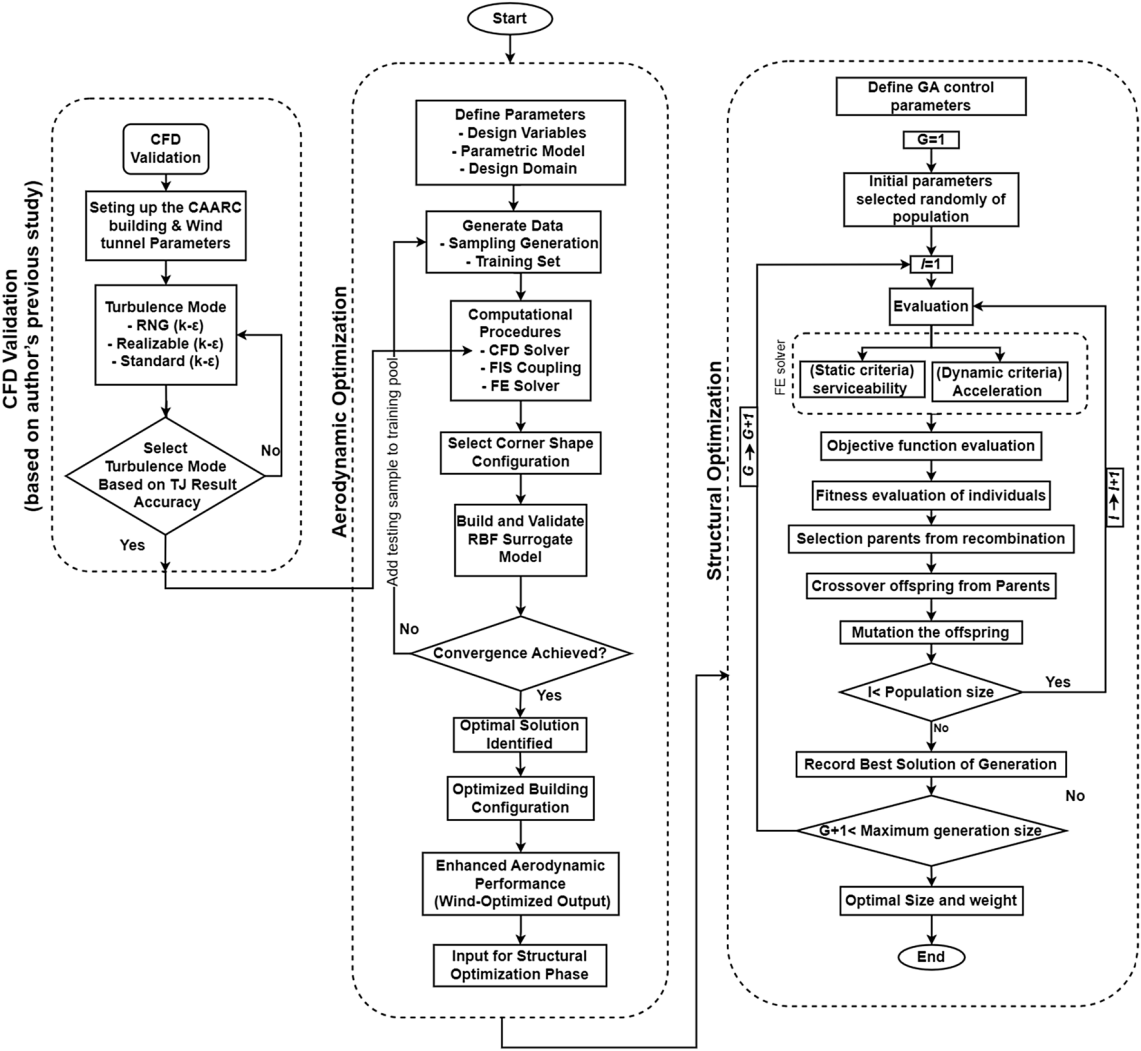
Integrated computational workflow for validated aerodynamic and structural optimization of super-tall buildings.

Mutation enhances crossover and selection by bringing stochastic changes to population solutions, enhancing algorithm optimization. Bit flip mutation, a critical operation in binary encoded GAs, randomly modifies offspring chromosome bits, introducing exploration and diversity. Figure 7 depicts GA development and structural optimization^45^.

## Optimization results and discussions

### Aerodynamic optimization

Aerodynamic optimization is conducted to optimize the corner configurations under wind load effect by utilizing the RBF surrogate model through the computational analysis processes, which are discussed in this section.

#### Maximum top displacement at designated sampling points for RBF surrogate modelling

**Fig. 8:**
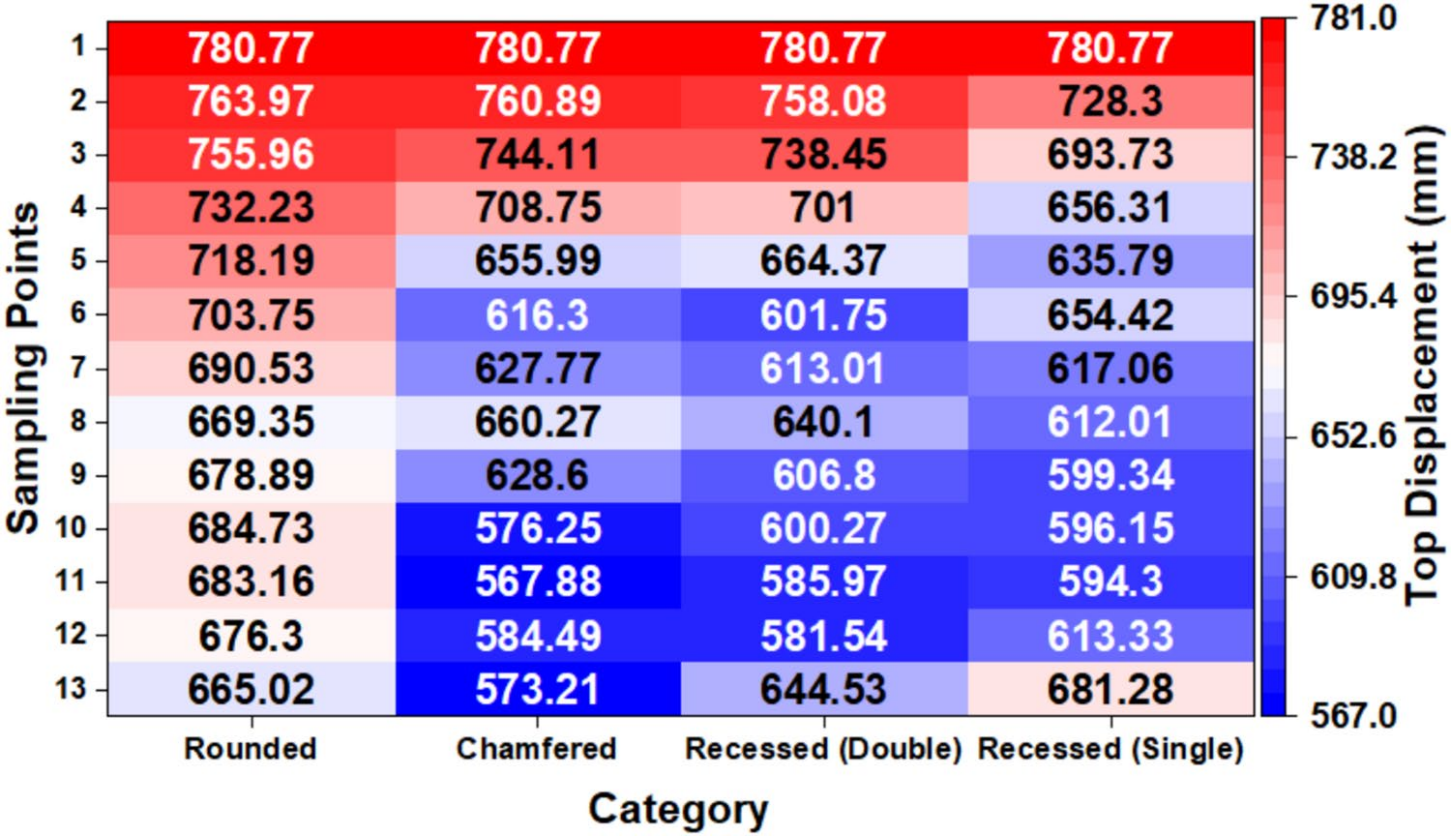
Heatmap of maximum top displacement of the chosen sampling points in different corner modification types.

Figure 8 shows the maximum top displacement at the selected sample points for different types of corner modifications. The rounded configuration generally results in the highest displacement, while the chamfered and recessed (double) configurations show a consistent reduction across most sampling points. The recessed (single) configuration provides moderate improvement compared to the rounded case. Then, these chosen sampling points are used to build the RBF surrogate model for optimizing the aerodynamics.

#### RBF surrogate model validation

**Fig. 9:**
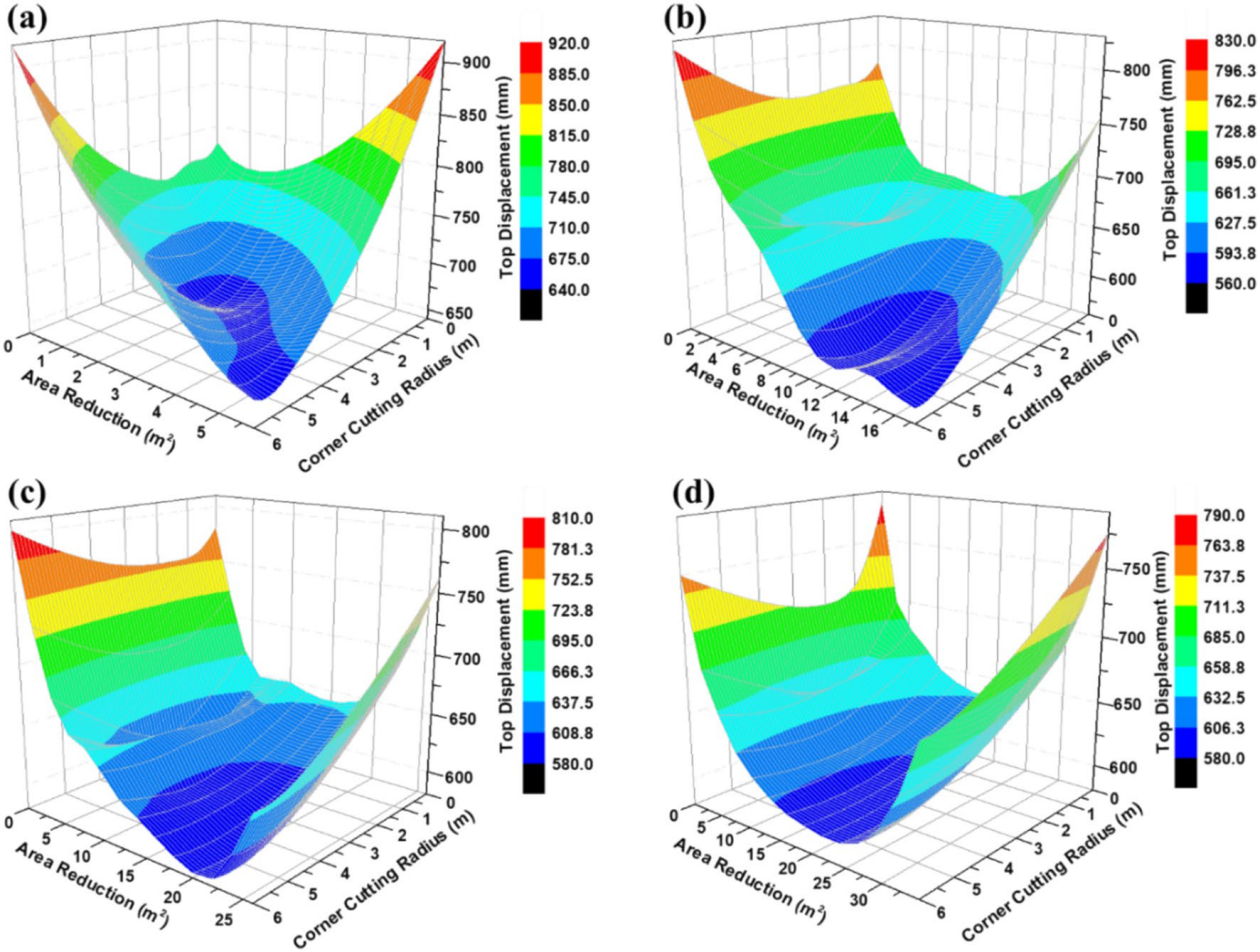
Prediction of total top displacement based on RBF under corner modifications and radius for various types of (**a**) rounded, (**b**) chamfered, (**c**) recessed double, and (**d**) recessed single.

The final surrogate model is depicted by the RBF surface presented in Fig. 9. The surface response reveals that the most promising optimal solutions are located within the blue-contour area. Consequently, the minimum top displacements for various corner modification types utilizing the surrogate RBF optimization algorithm are compiled in Table 3. The optimal value is associated with a large corner-cutting radius and significant area reduction. Conversely, the region linked to minimal corner-cutting radius yields the highest top displacement values. The optimal value derived from 13 samples is illustrated in the chamfer type. This aligns with established research indicating that chamfering building corners effectively mitigates wind-induced loads and enhances overall structural performance^55^.

**Table 3:** RBF optimization derived from 13 samples for different corner modification type and radius.

Type	Corner cutting radius, R (m)	Area reduction (m^2^)	Top displacement (mm)
Rounded	5.6	5.76	659.80
Chamfer	4.7	10.63	566.46
Recessed double	5.2	19.94	580.63
Recessed single	5.2	26.86	600.18

An additional sampling point resulting from the 13 samples was incorporated in the subsequent stage for the chamfer type to achieve the optimization value with an acceptable root mean square error, as detailed in Table 4.

**Table 4:** RBF optimization derived from 14 samples for chamfer corner modification and radius.

Predicted (RBF)	Actual (CFD-FSI-FE)		RMSE (%)
566.22	565.34	0.16	

### Structural optimization

The optimal design computation was conducted using a high-performance computer equipped with an Intel (R) Core (TM) i7-9750H CPU running at 2.60 GHz and 32.0 GB of RAM. The entire optimal design process took approximately 98 h to complete the runs for 50 generations with a population of 50. For every generation, the algorithm ranks and records the fittest individual within the population. This approach ensures that the best solution found so far is retained for the next generation if no better solution is discovered. Only generations with altered individual solutions are displayed.

#### Design constraints evaluation

**Fig. 10:**
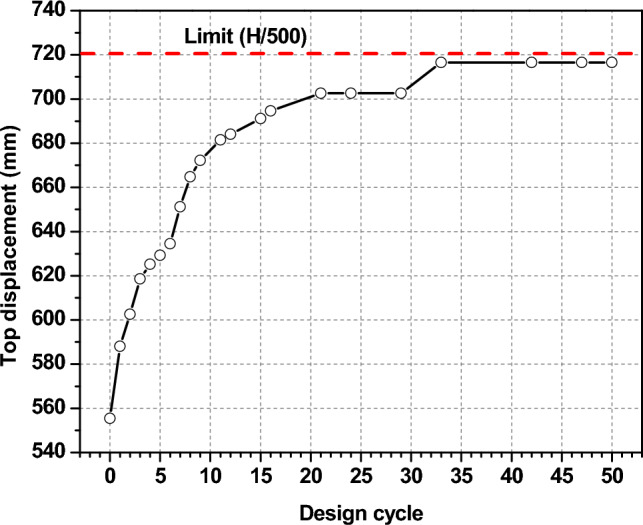
Top lateral displacement.

The design constraints that govern the optimization in this study were specified for each proposed individual solution during the GAs process (Fig. 7). Figure 10 shows how top lateral displacement has changed during optimization process cycles, which is an important part of the design strategy. It shows maximum increment in top lateral displacement, which increases to its optimal value at the final convergence, at the average to be a bit less than the limitation value.

**Fig. 11:**
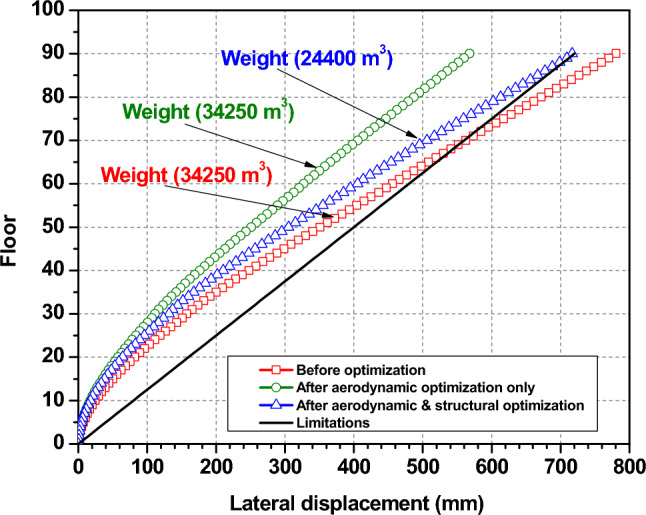
Lateral displacement before and after the optimization.

Before the optimization, top lateral displacement even exceeded the limitation stipulated in the code after three-fourths of the building’s height. This could be because the stiffness decreased with height. (i.e., floors 68 to 90). After the aerodynamic optimization due to advancements in aerodynamics, top lateral displacements are significantly reduced from 0.781 to 0.563 m, about a 28% improvement, and satisfy the code requirement of H/500 (0.720 m)^47^. This improvement illustrates that aerodynamic modification is an effective way to reduce the overall wind-induced lateral response, even if the structure’s stiffness remains unchanged. Then, after the structural optimization, it gradually grew throughout later design cycles to reduce the building weight as much as it could while maintaining the structural integrity, reaching 0.717 m as shown in Figs. 10 and 11, which means that top lateral displacements are the key constraint for this wind-resistant optimal design.

**Fig. 12:**
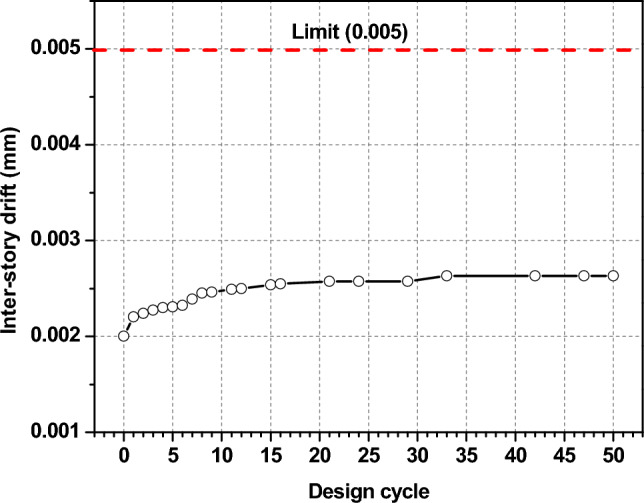
Maximum inter-story drifts.

Figure 12 demonstrates the maximum inter-story drift ratio changes before and after the optimization process, and it can be noticed that there is an increase of maximum inter-story draft ratio with height for all cases. The maximum inter-story drift ratio changes from 0.0027 to 0.002. This decrease shows the effectiveness of the aerodynamic modifications in mitigating wind load. However, upon structural optimization, the maximum inter-story drift ratio goes back up, getting closer to the level it was at before optimization. Nevertheless, all the three cases are always far smaller than the limits specified by the design code throughout the optimization process. This means that the maximum inter-story drift ratio is not a key factor in this optimal design.

**Fig. 13:**
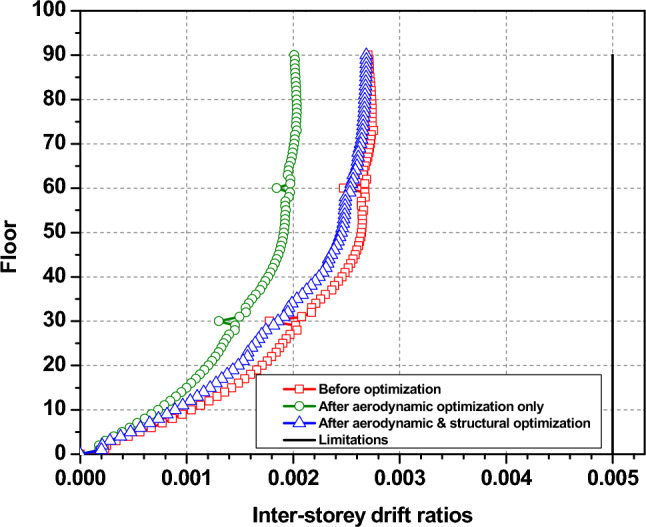
Inter-story drift ratios before and after the optimization.

In contrast, Fig. 13 presents the pre- and post-optimization changes in inter-story drift ratios, emphasizing minimal influence on the wind-resistant optimal design. Post-optimization, adjustments aimed at aerodynamic enhancements significantly reduce these ratios, ensuring compliance with stipulated criteria.

**Fig. 14:**
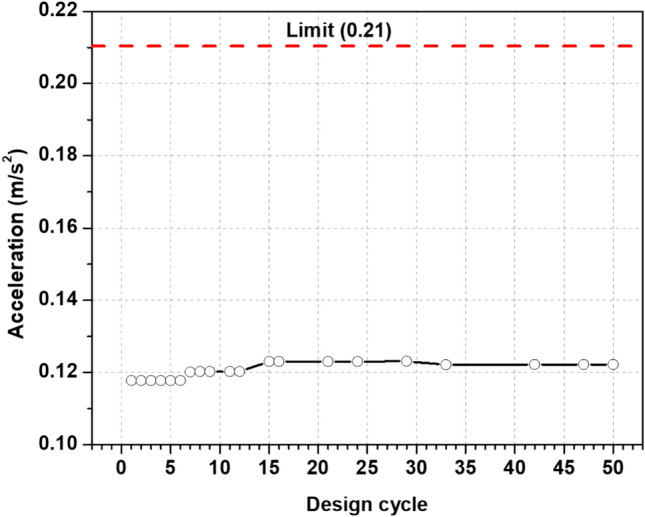
Top acceleration for critical mode of shapes.

Additionally, Fig. 14 shows the maximum top acceleration in the optimal design process cycles. It is indicated that the maximum top acceleration slightly increases until it reaches 15 design cycles to have an almost constant reading of about 0.12 m/s^2^. Overall, the reading throughout the operation shows that the maximum top acceleration is below the limit of 0.21 m/s^2^, which shows that the optimization technique works to manage wind-induced dynamic response and keeps the occupants comfortable.

#### Structural member optimization under lateral loading

**Fig. 15:**
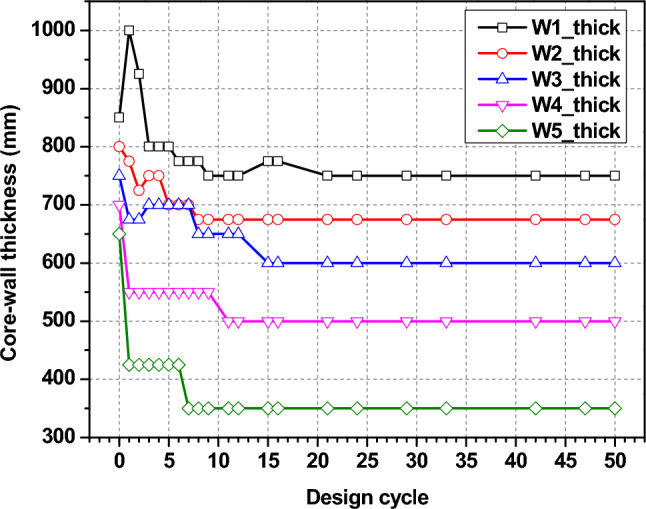
Wall section optimization.

**Fig. 16:**
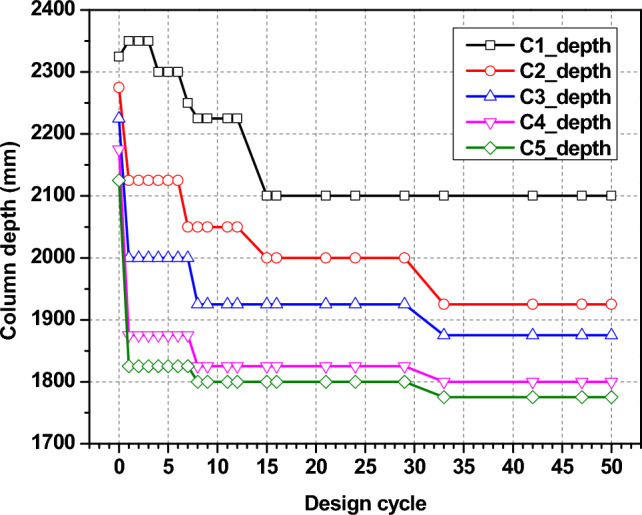
Column section optimization.

**Fig. 17:**
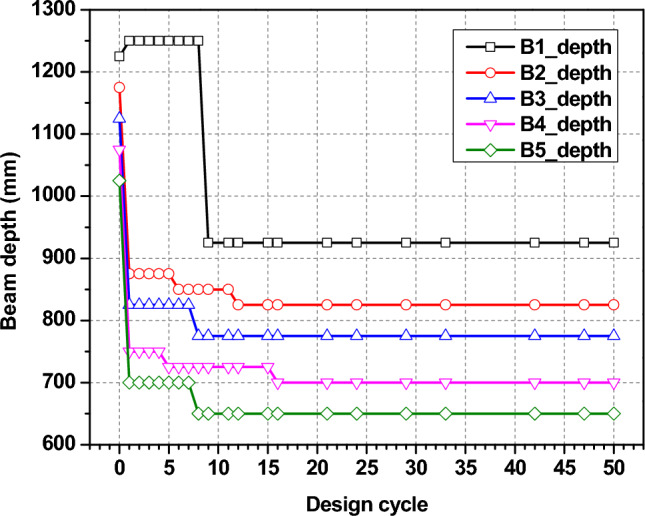
Beam section optimization.

An optimum solution is created by assembling predefined structural components for various structural elements. The core-wall sections (Fig. 15) show an initial decrease in core wall size, but after 15 optimization cycles followed by stabilization of the section size, illustrating the optimization method successfully reduces the wall thickness as much as possible without compromising structural integrity. Similarly with column and beam sections as shown in Figs. 16 and 17, respectively. The reduction in both the section size of the column and the beams throughout design cycles indicates a design adjustment to minimize material usage while meeting goals for performance. All these graphs show the iteration of the structural optimization, where all the structural components are adjusted to meet the structural performance and economic criteria, which makes sure the design is as optimal as possible.

#### Optimization output

**Fig. 18:**
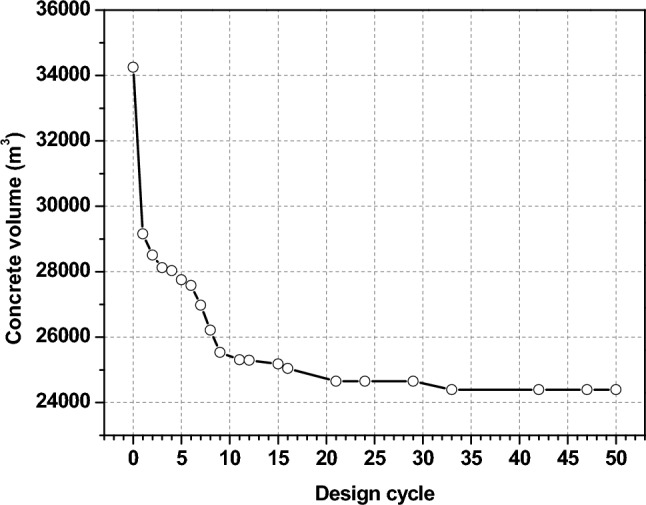
Optimal design history of the total volume and generation design cycle.

Figure 18 illustrates the amount of concrete needed for the lateral structural system, which was figured out by minimizing weight for each solution that was made. The first population was random; thus, the optimization method may easily discover the optimum solution with the fewest sections, which would make the structure significantly smaller. After that, selection and evaluation algorithms improve the solutions until they all have the same section sizes and the structure is optimized.

The difference in concrete volume between the original design and the best section weight of lateral structural members, which cuts the volume by about 9850 m^3^, or 28.8% of the original sections as shown in Fig. 16. Sustainable concrete manufacturing emits about 470 kg of CO_2_ for every 1 m^3^ of high-strength concrete. So, the optimization reduced the cost of building materials and eliminated 4630 tonnes of CO_2_ emissions.

## Replication of results

The methodology and results presented in this study are designed to ensure replicability and transparency. The complete Python code (provided as supplementary material) allows researchers to replicate the optimization process and validate the findings. The code includes modules for wind-induced vibration response analysis, structural optimization using enhanced GAs, and dynamic updating of wind loads based on changes in structural dynamic properties. The case study, modeled after a 90-story octagonal reinforced concrete frame structure, demonstrates the practical application of the proposed methodology. Detailed descriptions of input parameters, such as geometric configurations, material properties, and wind load characteristics, are included in the manuscript and supplementary material. Key constraints, including top displacement, inter-story drift ratios, and peak accelerations, are explicitly defined to guide users in replicating the study. The computational framework utilizes penalty functions to handle implicit constraints and optimize the lateral structural system. The methodology achieves significant reductions in wind load effects (28.2%) and concrete volume (28.76%), demonstrating its robustness and efficiency. Researchers replicating the results should use the same computational environment and parameters to ensure consistency.

## Conclusions, implications and outlook

### Summary

This research focuses on the advancement in structural optimization, particularly as it pertains to 90-story octagonal super-tall buildings exposed to wind motions, which was the primary goal of this project. There are two main steps to the method that has been devised. The first step is the proposal of a suitable computational method for the evaluation of wind loads on octagonal super-tall buildings. This is accomplished by combining an algorithm for the interaction of fluid structures with a parametric model for CFDs in order to generate wind forces. In order to optimize the structure, the produced forces are input with the forces of gravity. Using a GA with an improved constraint function, this research develops structural optimization. Mathematical formulations of the improved design constraints are established, taking into consideration lateral displacement, drifts, and top acceleration. To compare the static and dynamic structure responses to the predefined objective function, FEA is applied to each solution that is developed.

### Implications of the study

The viability of this research work is validated by conducting an existing case study of an octagonal super-tall building. In order to replicate the wind effect on the building under study, the CFD model solely used steady and incompressible flow applied at a zero-degree angle of attack. The established methods successfully optimized the building’s lateral structural system. To test this out, we cut the concrete volume by 28.76% from the initial plan, decreasing carbon dioxide emissions by 4000 tonnes. When working with huge projects such as super-tall buildings, design engineers greatly benefit from the computational methodologies that have been employed, especially at the conceptual stage. These methodologies are economically efficient and lead to significant reductions in construction and material expenses.

### Recommendations for future research

Future research will be enhanced by the utilization of various CFD models, incorporating multiple flow types and mesh resolutions, to enhance the precision of recording wind dynamics on tall structures. Additionally, a more in-depth look at other evolutionary algorithms, including Neural Network and Practical Swarm, can be done to see how well they function for structural optimization challenges. Another idea is that, thanks to the parametric technique used in this study, a different kind of optimization can be created and added to the suggested framework. That involves optimizing the building’s aerodynamics to reduce the effects of wind on it and optimizing its topology. To assess various lateral structural systems for optimal wind resistance solutions. Future work may extend the proposed framework to evaluate structural performance under different scenarios. In multi-scenario hazard optimization that might cover variable return periods and wind directions and account for along-wind, crosswind, and torsional wind effects, SLS and ULS can be applied across all scenarios. Various codes, i.e., ASCE 7, EN1991-1-4, and NBCC, suggested drawing scenario envelopes and optimizing based on the critical values. In a performance-based design, a reliability-based robust approach may be more appropriate. In this approach, scenario coefficients are considered uncertain and plotted in a probability distribution curve, which can be solved using first- or second-order reliability methods (FORM or SORM). Similarly, due to growing interest in integrating optimal design to determine control strategies that reduce dynamic response, using devices such as tuned mass dampers and viscous dampers for multi-optimization has proven effective in minimizing wind and seismic vibrations.

## Data Availability

The data supporting the findings of this study are available on reasonable request from the corresponding author through the email address [abtalmasoodi@gmail.com ; [abt.almasoodi@hau.edu.ye](mailto:abt.almasoodi@hau.edu.ye)].
